# The rates of the major steps in the molecular mechanism of RNase H1-dependent antisense oligonucleotide induced degradation of RNA

**DOI:** 10.1093/nar/gkv920

**Published:** 2015-10-10

**Authors:** Timothy A. Vickers, Stanley T. Crooke

**Affiliations:** Department of Core Antisense Research, ISIS Pharmaceuticals, Inc. 2855 Gazelle Court, Carlsbad, CA 92010, USA

## Abstract

Antisense oligonucleotides (ASOs) are most commonly designed to reduce targeted RNA via RNase H1-dependent degradation, however kinetic parameters for ASO-mediated targeting and subsequent cleavage and degradation of RNA in living cells are poorly understood. In this manuscript we use an inducible minigene system to determine the time course of ASO activity in the cell. Estimates of the time required for the ASO to enter and traverse the cell, scan the target mRNA, bind the cognate site, recruit RNase H1 and initiate cleavage, are presented in the context of transcription and mRNA processing rates. Data are also presented which indicate that rates for RNase H1-dependent ASO-mediated degradation of the targeted RNAs are different for nuclear-retained versus RNAs exported to the cytoplasm and that the level of RNase H1 in the cell and cellular compartments is limiting to the rate of ASO activity. In both cellular compartments RNase H1 ASOs essentially double the endogenous rates of clearance of the target RNA. Overexpression of *Escherichia coli* RNase H1 or the presence of multiple cognate sites each further increase the rate of target RNA degradation.

## INTRODUCTION

Antisense oligonucleotide (ASO) mediated degradation of targeted RNAs has been broadly exploited as both a research tool and a platform to create human therapeutics (1). The most frequently employed antisense mechanism is RNase H1-dependent degradation of the targeted RNA (2,3). Human cells express two types of RNase H: RNase H1 and RNase H2. Human RNase H1 is active as a single peptide, whereas RNase H2 is a heterotrimeric enzyme (4,5). Both enzymes are thought to play a role in DNA replication and repair, but additional biological functions are likely for both. Both RNase H isozymes recognize an RNA–DNA heteroduplex and cleave the RNA strand, resulting in a 5′-phosphate on the product and release of the intact DNA strand. RNase H1 is the enzyme responsible for mediating the target RNA cleavage directed by ASOs containing four or more consecutive DNA nucleotides (6). Human RNase H1 binds to the RNA–DNA heteroduplex through an RNA binding domain located on the N terminus of the protein and cleaves the RNA 7–10 nt, approximately one helical turn, from the 5′-end of the duplex region.

The kinetics of ASO-mediated targeting and subsequent cleavage and degradation of RNA in living cells are poorly understood. In effect, ASOs designed to serve as substrates for RNase H1 are inhibitors of the intermediary metabolism of pre- and spliced mRNAs. To fully understand the molecular events resulting in ASO activity it is important to understand the rates of each step in the molecular mechanisms of ASOs in the context of the rates of transcription, RNA processing, transport and degradation.

In this manuscript we employ a novel minigene system that we have developed and studied extensively (7–9) to understand the time course of events that induce ASO activity in the cell. We present estimates of the time required for the ASO to traverse the cell membrane, scan the target mRNA, bind the cognate site, recruit RNase H1 and initiate cleavage, in the context of other cellular processes. We also show RNase H1 rates of degradation for ASO-targeted RNAs and the effects of various factors on the activities of RNase H1 designed ASOs. Since RNase H-dependent ASOs are active both in the nucleus and cytoplasm (8,10), we also sought to determine if there are differences in rates for nuclear-retained versus RNAs exported to the cytoplasm. In addition, we present data demonstrating differences in rates for an ASO targeting a single site versus multiply repeated sites and show that the level of RNase H1 in the cell is limiting to the rate of ASO activity.

## MATERIALS AND METHODS

### Preparation of antisense oligonucleotides

Synthesis and purification of phosphorothioate/2′-MOE or S-cEt oligonucleotides was performed using an Applied Biosystems 380B automated DNA synthesizer as described previously (11). All ASOs were ‘gapmers’ 16–20 nt in length with 2′-O-methoxyethyl (MOE) or constrained ethyl (cEt) (12) substitutions at the positions indicated in in Table 1.

**Table 1.** Sequences of ASO gap-mers used in the study

ASO no	Sequence	Target	2′ substitution
440282	TTTCCACCTTTGCCCAAGTC	SOD1 exon 5	MOE
440283	GTACTTTCTTCATTTCCACC	SOD1 exon 5	MOE
496218	TCCACCTTTGCCCAAG	SOD1 exon 5	cEt
480776	ACAGTTTATCTGGATCTTTA	SOD1 intron 4	MOE
398457	GGGTTCCCGAGGTGCCCAAT	GCGR	MOE

All RNase H-dependent ASOs used for target mRNA reduction were 16–20 bases in length. All linkages were phosphorothioate with 2′-*O*-methoxyethyl (MOE) or constrained ethyl (cEt) residues incorporated at the underlined positions.

### TET-inducible mini gene system

Construction of the SOD1 minigene, pcDNA_SOD1 and generation of stable minigene cell lines with or without integration of the plasmid pcDNA3.1-RHA for overexpression of *Escherichia coli* RNase H1 was described previously (7). A splice-defective mutant, pcSOD187M/TO, in which the native U1 consensus sequence, UG GUAAGU was converted to UG GUUGGG to prevent binding of U1 snRNP at the 5′ splice site, was generated by site directed mutagenesis using a QuikChange Lightning SDM Kit (Agilent Technologies) with primers W187F 5′-CATCATTGGCCGCACACTGGTGGTTGGGTTTCATAAAAGGATATGCATAAAAC-3′ and W187R 5′-GTTTTATGCATATCCTTTTATGAAACCCAACCACCAGTGTGCGGCCAATGATG-3 according to the manufacturer's protocol. Cell lines with a 20 nt target site for GCGR inserted in the SOD1 minigene as a single or 4× repeat were generated by site directed mutagenesis as described previously (9).

T-REx-293 cells were purchased from Invitrogen and cultured in Dulbecco's modified Eagle's medium (DMEM) supplemented with 10% fetal calf serum, 0.1 μg/ml streptomycin, 100 units/ml penicillin and 5 μg/ml blasticidin. Plasmids pcSOD1/TO and pcSOD187M/TO were transfected into T-REx-293 cells using Effectene transfection reagent according to the manufacturer's protocol (Qiagen). Cells in which the minigene was stably integrated were selected in DMEM media containing 250 μg/ml zeocin. Zeocin-resistant colonies were expanded then tested for induction of expression by tetracycline (TET) using qRT/PCR. Cell lines overexpressing *E. coli* RNase H1 were generated by stably incorporating the plasmid pcDNA3.1-RHA as described previously (7). The copy number (CN) of minigenes was determined for each cell line by TaqMan CN assay using a forward primer, reverse primer and FAM dye-labeled TaqMan MGB probe designed to recognize the pcDNA4/TO plasmid in which the minigene was cloned. TaqMan CN Assays were performed according to the manufacturer's instructions (P/Ns 4 400 294, Applied Biosystems, Foster City, CA, USA) with genomic DNA isolated from 5 × 10^6^ cells purified with a PureLink Genomic DNA Mini kit (Life Technologies). For each cell line, four replicate CT values were obtained per sample with FAM-labeled and VIC-labeled probes, assaying the promoter region of the pcDNA4/TO plasmid in which the minigene was cloned and an RNase P control gene (P/Ns 4 403 328, Applied Biosystems), respectively. CN was calculated by analysis of the polymerase chain reaction (PCR) results with Copy Caller software.FP-GTACGGTGGGAGGTCTATATAAGCA, RP-CGATCTGACGGTTCACTAAACGA, PRB-ATCACTGATAGGGAGATCTC

### Determination of minigene transcription, splicing and degradation rates

Transcription and splicing rates were determined by addition of 1 μg/ml TET to the SOD187M/TO cells (transcription) or SOD/TO cells (splicing) seeded in 96-well plates at a concentration of 10 000 cells/well in growth medium. Media was removed and cells lysed by the addition of RLT lysis buffer at specific intervals following addition of TET. Total RNA was purified using an RNeasy 96 Kit (Qiagen) and mRNA levels were assessed by qRT/PCR performed essentially as described elsewhere (13). Briefly, 10 μl of total RNA was analyzed in a final volume of 50 μl containing 200 nM gene-specific PCR primers, 0.2 mM of each dNTP, 75 nM fluorescently labeled oligonucleotide probe, 5 μl RT-PCR buffer, 5 mM MgCl_2_, 2 U of Platinum Taq DNA Polymerase (Life Technologies) and 8 U of RNase inhibitor. Reverse transcription was performed for 30 min at 48°C followed by PCR: 40 thermal cycles of 30 s at 94°C and 1 min at 60°C using an ABI Prism 7700 Sequence Detector (Applied Biosystems). To avoid artifacts based upon well-to-well variation in cell number, mRNA levels were normalized to the total amount of RNA present in each reaction as determined by the Invitrogen Ribogreen assay (14). Primers were designed to specifically amplify spliced or pre-mRNA and both were detected by the same probe. To avoid amplification of endogenous SOD1, primers included vector sequence unique to the mini gene (7). Absolute quantities of RNA transcribed were determined using standards of known concentration produced as previously described (7). The molecular weight of minigene RNA was calculated using a program developed by EnCor Biotechnology, Inc. (http://www.encorbio.com/protocols/Nuc-MW.htm), which takes a nucleic acid sequence and calculates the molecular weight and GC content.

To determine RNA degradation rates, cells were seeded in 96-well plates and transcription of the minigene induced to steady state levels by addition of 1 μg/ml TET for 16 h. The following day the cells were washed extensively with phosphate buffered saline (PBS) to remove TET and stop transcription, then treated with the indicated concentrations of ASOs in Opti-MEM media (Invitrogen) containing 5 μg/ml Lipofectamine 2000 (Invitrogen), as described previously (15). Transfections were terminated at various times after initiation of ASO treatment with RLT lysis buffer and total RNA purified using an RNeasy 96 Kit (Qiagen). Levels of target RNA were determined by qRT/PCR as above.

Degradation was also measured in experiments in which ASO treatment preceded minigene induction. Cells in 96-well plates were transfected for 4 h with ASO using lipofectamine 2000 as detailed above. Following the transfection, cells were washed 1× with PBS, then DMEM + TET added to initiate transcription. Transcription was stopped by addition of RLT lysis buffer at the indicated time points. RNA was then purified and levels of target RNA were determined by qRT/PCR.

## RESULTS

We have previously reported construction of a minigene comprised of exon 4, truncated intron 4 and exon 5 of the SOD1 gene (7) and a splice-defective mutant, pcSOD187M/TO, in which the native U1 consensus sequence was mutated prevent binding of U1 snRNP (8). We took advantage of the low levels of splicing in the SOD187M/TO cell line to analyze the minigene transcription rate. Minigene transcription was induced in both control and splice-defective cell lines by addition of TET to the growth media. Cells were harvested at specific intervals following addition of TET, then minigene expression assessed by qRT/PCR using a primer/probe specific to the pre-mRNA. Absolute quantities of RNA transcribed were determined using standards of known concentration in the qRT/PCR reaction.

**Figure 1.**
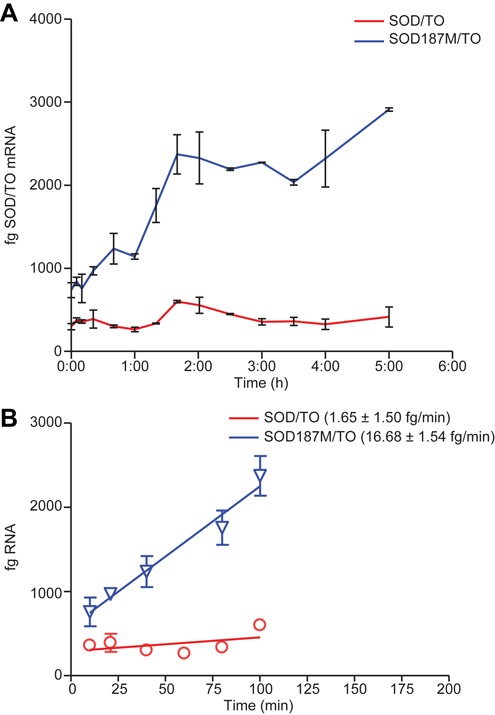
SOD1 minigene transcription rate. Minigene expression was induced by addition of TET to 10^5^/well SOD/TO or SOD187M/TO cells in 96-well plates. At specified times post-induction, cells were harvested and total RNA purified. About 1/10th of the total RNA was subject to qRT/PCR utilizing a primer/probe set specific to the minigene pre-mRNA. Absolute quantities of RNA were obtained by running a standard curve in the reaction with minigene pre-mRNA of known quantity prepared *in vitro* using T7 RNA polymerase. (**A**) Femtograms of pre-mRNA from SOD187M/TO cells (blue) or SOD/TO cells (red) per qRT/PCR reaction, representing total RNA input from 1000 cells. (**B**) Data in panel A plotted as linear regression from 5–100 min.

For the SOD/TO control minigene, relatively little pre-mRNA was detected (Figure 1, red), presumably due to the rapid splicing of the mingene intron. In contrast, increased levels of the pre-mRNA transcript were detectable within 5 min of the addition of TET to the SOD187M/TO cell line and plateaued at ∼100 min (Figure 1A, blue). The data, plotted as a linear regression from 5–100 min, show that the pre-mRNA accumulated at a rate of ∼16.7 fg SOD pre-mRNA/min based on the qRT/PCR reaction (Figure 1B). Given that the total RNA input into the qRT/PCR reaction was from 1000 cells, the SOD pre-mRNA accumulates at a rate of 16.7 ag min/cell. The 567 nt SOD minigene pre-mRNA has a molecular weight of 168.14 KDa or 2.92 E-19 g which corresponds to roughly 60 transcripts/min, or a transcription rate of 34 kB/min. The CN of the minigene in this cell line was determined to be 6 (Supplemental Figure S1), therefore the transcription rate is ∼5.7 kb/min.

**Figure 2.**
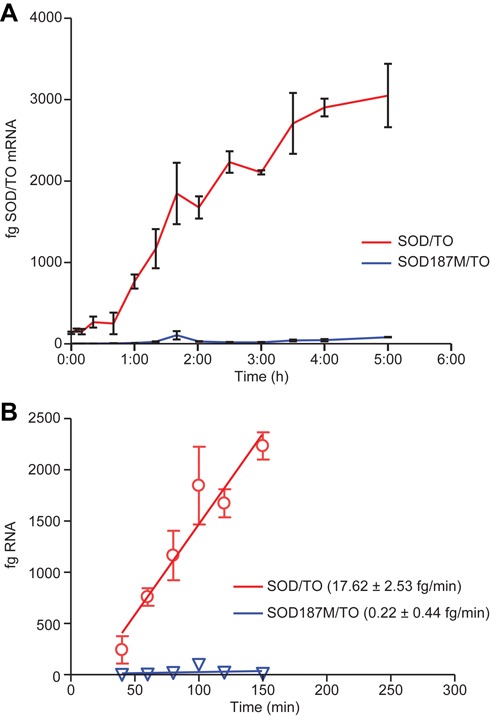
SOD1 minigene RNA processing rate. Minigene expression was induced by addition of TET to 10^5^/well SOD/TO or SOD187M/TO cells in 96-well plates. At specified times post-induction, cells were harvested and total RNA purified. About 1/10th of the total RNA was subject to qRT/PCR utilizing a primer/probe set specific to the spliced minigene mRNA. Absolute quantities of RNA were obtained by running a standard curve in the reaction with minigene spliced mRNA of known quantity prepared *in vitro* using T7 RNA polymerase. (**A**) Femtograms of pre-mRNA from SOD/TO cells (red) or SOD187M/TO cells (blue) per qRT/PCR reaction, representing total RNA input from 1000 cells. (**B**) Data in panel A plotted as linear regression from 30–150 min.

The splicing rate of the minigene was similarly estimated using the SOD/TO control minigene stably incorporated into TREX 293 cells. Again, minigene transcription was induced by addition of TET to the growth media. Cells were harvested at specific intervals following addition of TET, then minigene expression assessed by qRT/PCR using a primer/probe specific to the spliced mRNA. Absolute quantities of RNA were determined by using standards of known concentrations. For the pcSOD187M/TO control minigene almost no spliced mRNA was detected (Figure 2, blue), again demonstrating the effectiveness of the splice site mutation in reducing processing efficiency of the primary transcript. For the SOD/TO cell line, levels of the spliced mRNA transcript became detectable ∼30 min following addition of TET and began to plateau after ∼ 3 h (Figure 2A, red). The data, plotted as linear regression from 30–150 min, indicate that the spliced RNA accumulates at a rate of ∼17.6 fg SOD mRNA min^−1^ in the reaction (Figure 1B). The spliced mRNA is 304 nt in length which is 0.6× the length of the pre-mRNA. However, in this cell line we determined that there were nine copies of the minigene (Supplemental Figure S1) as compared to six copies in the mutant cell line or 1.5× more transcripts. On a per cell basis, the accumulation rate is therefore 17.6 × 0.6 × 1.5 = 15.8 ag/min/cell which is very close to the accumulation rate of the pre-RNA in the SOD187M/TO line, and is equivalent to the transcription rate minus the intrinsic nuclear degradation rate. As one would expect, spliced RNA accumulates in the cell at a rate similar to that of the pre-mRNA because transcription and splicing rates are closely matched (16).

The endogenous degradation rates in the nucleus and cytoplasm were determined and compared with the rates of degradation in the presence of ASOs. To measure mRNA degradation rates, SOD/TO cells were induced overnight with TET. The following day the cells were washed extensively, then transfected with ASO 440283, which is complementary to a sequence in exon 5 of the minigene. Total RNA was purified at various times after the initiation of transfection and levels of spliced mRNA determined by qRT/PCR. For nuclear degradation rates, SOD187M/TO cells were treated with ASO 480776, which is complementary to sequence within the minigene intron.

**Figure 3.**
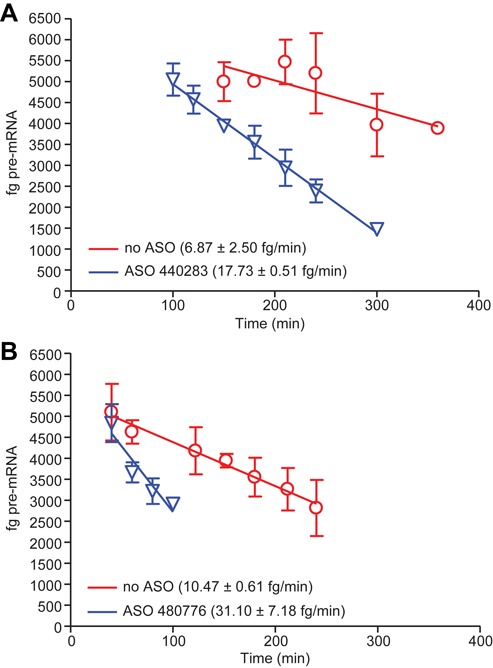
Nuclear and cytoplasmic minigene RNA degradation rates. (**A**) HEK 293 cells harboring the SOD/TO minigene were induced overnight with Tet to establish steady-state RNA levels. After removing TET and washing, cells were transfected with ASO, then harvested at the indicated times (min). Levels of total minigene spliced RNA were assessed by qRT/PCR as described in Figure 2. Data is presented as the linear regression from 120–360 min for mock transfected (red lines) or 90–300 min for cells treated with 50 nM ASO 440283 (blue lines). (**B**) HEK 293 cells harboring the SOD187M/TO minigene were induced overnight with Tet to establish steady-state RNA levels. After removing TET and washing, cells were transfected with ASO 480 776 at 50 nM then harvested at the indicated times (min). Levels of total minigene pre-mRNA were assessed by qRT/PCR as described in Figure 1. Data are presented as the linear regression from 20–240 min for mock transfected (red lines) or 20–100 min for cells treated with ASO (blue lines).

In the absence of ASO, the decay rate of the mRNA was determined to be roughly 6.9 fg/min whereas the decay rate for the pre-mRNA was ∼1.5-fold faster at 10.5 fg/min (Figure 3, red lines). RNA half-lives were also determined by arresting transcription with actinomycin D (Supplemental Figure S2). Consistent with the decay rates, the half-life of the mingene mRNA in cytoplasm was ∼1.5× longer than in the nucleus. Transfection of cells with an ASO complementary to the minigene resulted in a significant increase in the rate of degradation. For the spliced mRNA, degradation increased to a rate of 17.7 fg/min while degradation of the pre-mRNA was more rapid with a rate of 31.1 fg/min. Together these data suggest that RNA degradation may occur more rapidly in the nucleus than in the cytoplasm (Figure 3B) and that an ASO designed to serve as a substrate for RNase H1 approximately double the normal degradation rate of the RNA in both the cytoplasm and nucleus.

### Effect of RNase H1 levels on degradation rates

**Figure 4.**
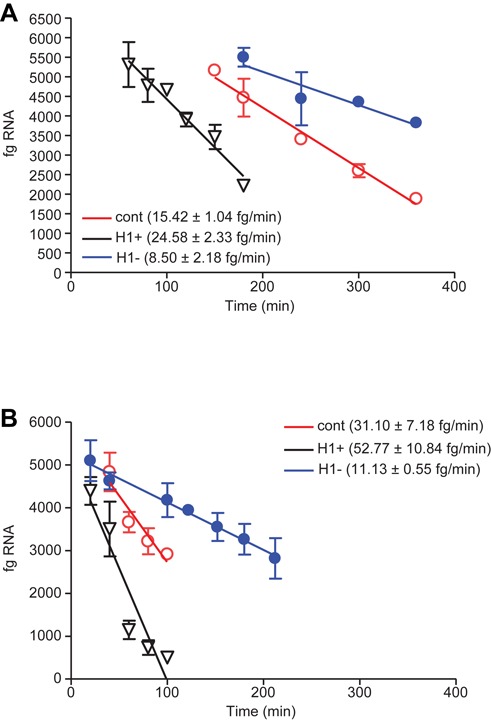
Effect of RNase H1 levels on ASO induced degradation rates. (**A**) SOD/TO cells were treated 24 h with an siRNA targeting RNase H1. SOD/TO control (cont), RNase H1 overexpressing (H1+) or RNase H1-reduced (H1−) cells were induced overnight with Tet to establish steady-state RNA levels. Cells were transfected with 50 nM ASO 440282 then harvested at the indicated times. Levels of total minigene spliced RNA were assessed by qRT/PCR as described in Figure 2. Data is presented as the linear regression from 120–360 min for control cells (red lines) or 180–360 min for RNase H1-reduced cells (blue lines) or 60–180 min for RNase H1 overexpressing cells (black lines). (**B**) Control, RNase H1 overexpressing or RNase H1-reduced cells as above, but with the SOD187M/TO cell line. Data is presented as the linear regression from 30–100 min for control cells (red lines) or 30–240 min for RNase H1-reduced cells (blue lines) or 10–100 min for RNase H1 overexpressing cells (black lines).

We next evaluated the effect of reduced RNase H1 levels on the rate of degradation. To increase levels of RNase H1, minigene cell lines in which *E. coli* RNase H1 was over expressed were utilized (7). To reduce levels of RNase H1 in cells, the SOD/TO line was treated with an siRNA targeting RNase H1 which was determined to reduce expression of RNase H1 by ∼80% by qRT/PCR (data not shown). After allowing 24 h for reduction of the protein, cells were seeded in 96-well plates and minigene expression induced with Tet to steady state levels overnight. Cells were then washed and treated with an ASO targeting the minigene. In control cells, the target RNA was degraded at a rate of 15.4 fg/min (Figure 4, red line), similar to our previous observations. RNase H1 overexpression (black line) increased the degradation rate significantly to 24.6 fg/min, while reduction of RNase H1 (blue line) had the opposite effect, reducing the rate to 8.5 fg/min, which is very near the cytosolic degradation rate in the absence of ASO. Similarly, the rate of ASO-mediated pre-mRNA reduction nearly doubled with overexpression of RNase H1, increasing from 31.1 to 52.8 fg/min and was reduced to 11.1 fg/min in RNase H1 siRNA treated cells, approximately the same as the native nuclear degradation rate.

### Degradation rate is significantly increased at multiple repeat sites and is further enhanced by overexpression of RNase H1

**Figure 5.**
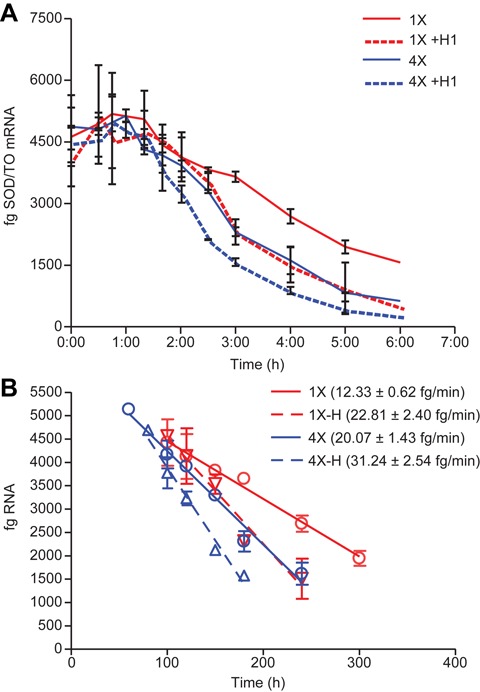
Comparison of the effect of repeated target sites and RNase H1 overexpression on ASO induced degradation rates. *Escherichia coli* RNase H1 was stably overexpressed in T-REx-293 cells harboring the SOD1- minigene containing one or four repeat sequences from GCGR. Minigene cell lines +/− *E. coli* RNase H1 were induced overnight with TET, then transfected with 50 nM ASO 398457. Minigene mRNA reduction was assessed by qRT/PCR at the indicated times as in Figure 2. (**A**) Femtograms of spliced mRNA from SOD/TO-GCGR 1X cells (red) or SOD/TO-GCGR 4X cells (blue) per qRT/PCR reaction, representing total RNA input from 1000 cells. Dashed lines are from cells overexpressing RNase H1. (**B**) Data in panel A plotted as linear regression from 60–300 min.

We have previously reported that the rate of mRNA degradation caused by RNase H1-dependent ASOs is increased in transcripts with repeated target sites (9). To determine the effect of RNase H1 levels on activity at repeated sites, transcription was induced overnight with TET in cell lines stably harboring SOD/GCGR-1X or SOD/GCGR-4X minigenes. These cell lines have a target site from an exogenous gene, GCGR, inserted in exon 4 of the SOD1 minigene as a single site or as four repeats of the same site. The next morning cells were washed to remove TET, then transfected with 50 nM ASO 398457, an ASO targeting the GCGR inserted sites. Cells were harvested at various times following initiation of ASO treatment and RNA reduction was assayed by qRT/PCR. As previously observed, target RNA reduction was not detected until 1.5–2 h after the initiation of transfection (Figure 5). For the cells expressing the transcript with the four repeat sequences, the rate of degradation was approximately two-fold faster than for the transcript with a single site (compare solid lines). Overexpression of RNase H1 had a similar effect on the rate of degradation of the cell line with the single site also increasing the degradation rate about two-fold (red lines). A combination of repeated sites and RNase H1 overexpression resulted in a further increase in the rate of degradation nearing three-fold that of the single site.

### Estimation of the times required for steps prior to RNase H1 cleavage

**Figure 6.**
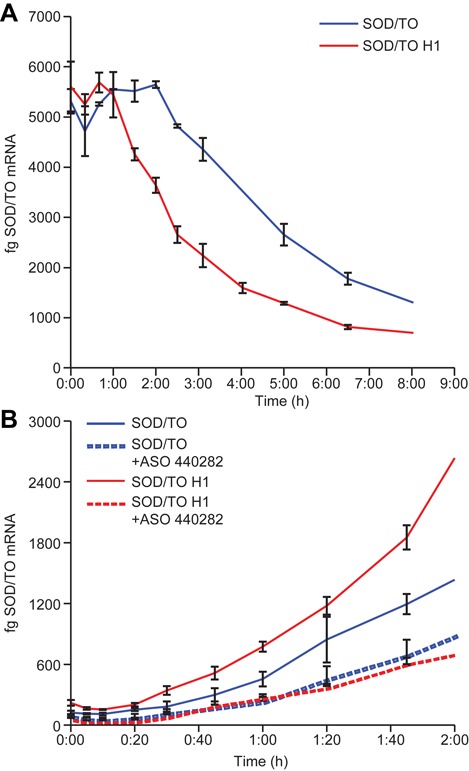
The effect of RNase H1 levels on the timing of the steps in the ASO-induced RNase H1 cleavage of a targeted RNA. (**A**) Estimates of ASO transit and RNase H1 recruitment times. HEK 293 cells harboring the SOD/TO minigene (red lines) or the SOD/TO minigene and pcDNA3.1-RHA (blue lines) were induced overnight with Tet to establish steady-state RNA levels. Tet was removed and cells washed extensively to stop transcription. Cells were immediately transfected with ASO 440 282 using Lipofectamine 2000, then harvested at various times after initiation of transfection with RLT lysis buffer and total RNA purified. Levels of total minigene RNA were assessed by qRT/PCR as described in Figure 2 for control (blue) and RNase H1 overexpressing (red) cell lines. (**B**) ASO Pre-load. HEK 293 cells harboring the SOD/TO minigene or the SOD/TO minigene and pcDNA3.1-RHA were transfected for 4 h with ASO 440282. ASO was removed and cells washed, then incubated in DMEM + TET to initiate transcription. Cells were harvested at various times after initiation of transcription with RLT lysis buffer and total RNA purified. Levels of spliced minigene RNA were assessed by qRT/PCR. Solid lines, mock transfected control; dashed lines, 25 nM ASO 440 282.

Several studies have demonstrated that the low cellular concentration of RNase H1 is rate limiting for the activity RNase H1-dependent ASOs (6,9). We previously reported that the rate of ASO-induced cleavage in cellular extracts prepared from cells overexpressing *E. coli* RNase H1 is increased by over 100-fold (7). Elimination of RNase H1 as the rate-limiting step by overexpression of *E. coli* RNase H1 provides an opportunity to estimate the time required for ASO transit, scanning, cognate sequence binding and RNase H1 complex recruitment. Minigene expression was induced to steady state levels in control and RNase H1 overexpressing cell lines by addition of 1 ug/ml TET for 16 H. Cells were washed extensively, then treated with 50 nM ASO 440282. Target mRNA expression was monitored at given time points post ASO transfection by qRT/PCR. In control cells expressing normal levels of RNase H1, reduction in mRNA was not observed until ∼2 h after the initiation of transfection (Figure 6A, blue line). This represents the time for the ASO to traverse the cell, scan the sequence, bind the cognate site, recruit RNase H1 and observe initial degradation. With overexpression of RNase H1, initial degradation was observed 1 h earlier (Figure 6A, red line). Since RNase H1 is not rate limiting in this cell line, this represents the time required for the ASO to enter and distribute throughout the cell and bind to its cognate site following initiation of transfection and suggests that another hour is required for the ASO bound site to recruit RNase H1 and initiate degradation of the target. To evaluate the effect of ASO length and chemistry on transit time and degradation rates, cells were treated with ASO 496218, a 16-mer cEt gapmer targeting the same site. ASOs containing the cEt modification have previously been shown to have greater affinity for the target RNA, thus allowing for comparable binding with shorter ASO length (12). The degradation kinetics of ASO 496 218 were similar to those for the MOE gap-mer, 440282 (Supplementary Figure S3), suggesting that ASO length and chemistry have little effect on cellular transit times and that degradation rates are primarily determined by the levels of RNase H1.

These data were confirmed by pre-loading SOD1 minigene cell lines with ASO. Cells were treated for 4 h with 50 nM ASO 440282. ASO was then removed and replaced with complete media plus TET to initiate transcription. Cells were harvested and total RNA purified at various times after initiation of transcription and RNA levels measured by qRT/PCR. In control SOD/TO cells without RNase H1 overexpression, ASO treatment resulted in reduction in the amount of the minigene mRNA that became observable at ∼60 min after the initiation of transcription (Figure 6B, red lines). Since uptake and intracellular distribution of ASO preceded minigene induction, this period only includes the time required for the ASO to bind its’ cognate site, recruit RNase H1 and initialize degradation. In SOD/TO cells with RNase H1 overexpression, only 20–30 min were required to observe ASO-mediated reduction of the mRNA (blue lines). Since RNase H1 is not rate limiting in these cells, this period is an estimate for time required for ASO to scan sequence and bind cognate the cognate site. The 30–40 min difference without RNase H1 overexpression is therefore an estimate of the time required to recruit RNase H1, and initialize degradation.

## DISCUSSION

To evaluate kinetic parameters associated with ASO-mediated degradation of RNA, we utilized a TET inducible minigene system. Our characterization of RNA transcription and splicing rates appears to be consistent with previously published data. These studies have used a range of methods to calculate the rate of Pol II elongation (16). Measurement of the Pol II elongation rate in mammals has been subject to substantial variation, with estimates between 1.3 and 4.3 kb/min. While our calculated rate is higher, it should be noted that, as in our experiments, the upper end of the range was also obtained using an engineered gene cassette (17). In another report in which qRT/PCR was used to analyze the rate of transcription of endogenous genes, the rate was determined to be 3.8 kb/min (18). It is not surprising that, using the same method in combination with a very short and highly optimized minigene, even more rapid transcription rates were observed. More recently, transcription rates above 50 kb/min have been reported in living cells (19), which points toward a wide dynamic range of RNAPII velocities. In the absence of ASO treatment, the transcription rate and the rate of accumulation of the spliced minigene were nearly the same (Figure 1/2), again suggesting the accuracy of these numbers.

We have previously shown that following transcription, the control SOD1 minigene mRNA is rapidly transported to the cytoplasm, while the SOD187M/TO minigene RNA is inefficiently processed and, as a result, remains confined to the nucleus (8). In our system, the transcription rate of the SOD187M pre-mRNA was 2.78 ag/min/copy/cell while the processing rate for the SOD/TO spliced mRNA was 1.95 ag/min/copy/cell. Based upon these rates, and assuming that the steady-state mRNA level is the result of transcription rate versus mRNA degradation, one would expect the rate of degradation to be faster in the nucleus than in the cytoplasm. Using these cell lines and qRT/PCR sets specific for the spliced or pre-mRNA, we were able to determine degradation rates for nuclear and cytoplasmic RNA. In the absence of ASO, the rate of degradation in the cytoplasm was ∼1.5-fold less than that observed in the nucleus (Figure 3). Consistent with this, the half-life for the spliced mRNA following treatment with actinomycin D was determined to be ∼1.5-fold that of the pre-mRNA (Supplementary Figure S2).

It has previously been reported that the half-life for certain transcripts is similar in the nucleus and cytoplasm (20). However, many pathways exist for RNA degradation (21) which may influence the overall rate of degradation of a particular message. During normal RNA synthesis, mRNAs are transcribed, spliced, capped and polyadenylated in the nucleus, then the resulting RNA is transported from nucleus to cytoplasm via nuclear pore complexes for translation. When export fails, as is the case with the SOD187M/TO minigene, nuclear pre-mRNA decay occurs via both 3′-to-5′ and 5′-to-3′ degradation pathways. The main actors in nuclear mRNA degradation are the exosome and Xrn2, which are responsible for 3′-to-5′ and the 5′-to-3′ exonuclease activity respectively (22). In the cytoplasm the degradation of stable transcripts is normally initiated by removal of the poly(A) tail followed by 5′-cap hydrolysis and degradation of the remaining mRNA by Xrn1. Alternatively, the exosome complex degrades mRNA in the 3′-to-5′ direction. Faulty transcripts may also be degraded via nonsense-mediated decay (23). It is not surprising that these different mechanisms would result in different rates of degradation. For example, unlike cytoplasmic mRNAs which are stabilized by polyadenylation, nuclear RNAs may be destabilized via polyadenylation by the TRAMP complex, leading to accelerated exosome-mediated 3′-to-5′ decay (24).

Treatment of cells with RNase H1-dependent ASOs resulted in significant increases in the rate of degradation of the minigene RNA both in the nucleus and cytoplasm (Figure 3, blue lines). In the nucleus, treatment with ASO resulted in a three-fold increase in the rate of degradation, whereas in the cytoplasm the degradation rate was increased 2.6-fold. These data are consistent with previous reports that ASOs targeting nuclear-retained RNAs are, in general, more potent than those targeting cytoplasmic RNAs (25). It is also known that RNase H1 is more highly expressed in the nucleus than the cytoplasm (26). It is therefore likely that ASO-induced degradation rates are dependent on the local concentration of RNase H1 and that once cleaved, the RNA degradation machinery rapidly processes the resulting fragments, presumably with the 5′ mRNA fragments degraded from their 3′ ends by the exosome and the 3′ fragments degraded from their 5′ ends by XRN1/2 (27,28).

When RNase H1 was reduced in the cell, ASO-induced degradation rates of the mingene RNA were almost the same as in the absence of ASO (compare Figures 3 and 4). Conversely, when RNase H1 was overexpressed, the onset of degradation was accelerated and rates were nearly doubled (Figure 4, black lines), confirming that the cellular concentration of RNase H1 is rate limiting for ASO activity. We have previously shown that degradation is significantly enhanced at multiple repeat sites, with the increase in activity at repeated sites the result of increased ASO hybridization frequency and recruitment of RNase H1 to a particular RNA target, which increases the rate of target cleavage and degradation (9). In this study we compared the rates of degradation at single and multiple sites and also the effects of RNase H1 levels. The presence of a target site repeated four times or overexpression of RNase H1, had very similar effects, increasing the degradation rate about two-fold compared to the single site without RNase H1 overexpression (Figure 5). Decay rates were further increased by combining RNase H1 overexpression and the repeated sites.

We took advantage of the fact that RNase H1 is rate limiting to estimate the times required for the steps preceding ASO-mediated RNase H1 cleavage. For example, in the absence of RNase H1 overexpression, reduction in the spliced minigene was observed after ∼2 h (Figure 6A). It has been shown that lipid transfected siRNAs are released from maturing endosomes within 5–15 min of uptake and begin to be loaded into Ago2–RISC complexes after 30 min (29). In these same experiments, detectable reduction in targeted mRNA was not observed for at least 2 h. Thus, it is likely that only a small fraction of the time to observed degradation is required for the lipoplexed ASO to traverse the membrane. When RNase H1 was overexpressed and no longer rate limiting, target reduction was observed 1 h earlier, indicating that the ASO is able to traverse cell membrane, distribute and bind its cognate site within 60 min of the initiation of transfection. If this is the case, under conditions in which levels of RNase H1 are normal and after the ASO enters the cell, an hour is required to recruit RNase H1 and initiate target RNA degradation. It must be emphasized that these times are estimates, since it is possible that recruitment of RNase H1 may be even slower when present at normal cellular levels.

By loading the cell with ASO, prior to initiating transcription of the minigene, we were able to further elucidate the timing of the steps following uptake. Under these conditions, and in the presence of normal levels of RNase H1, reduction in the minigene was not observed for 50–60 min (Figure 6B, red lines), consistent with our previous observations. When the experiment was done in cells overexpressing RNase H1, reduction in the minigene RNA was observed by 20–30 min (blue lines), representing the time required to bind the target. The 30–40 min difference in time to observed reduction between the control and RNase H1 overexpressing cells includes recruitment of RNase H1 complex, cleavage and subsequent degradation of the RNA, following target binding by the ASO. These data once again emphasize the importance of the levels of RNase H1 in determining the rate of loss of targeted RNAs.

We considered exploring the rates of ASO activity following gymnotic administration, however it takes as long as 3–5 days before an effect is seen with free uptake of ASOs in cell culture, depending on the cell line being used (30). Certain cells lines have been found to take ASOs up more quickly and efficiently in the absence of lipid (31), however even in these cell lines, target reduction is not observed until 6–8 h after the initiation of treatment. Clearly, this is much slower than the timescale of the other processes we have reported. Moreover, even at high ASO concentration and with extended administration times, we found that TREx cells do not take up ASO at levels sufficient to reduce the targeted RNA (data not shown). More importantly, with lipoplexed ASO, the uptake rate is quite rapid relative to other rates and we can zero uptake time by performing the transfection before inducing transcription. With gymnotic administration, uptake becomes rate limiting. As a consequence, it is difficult to assess other rates that are the primary focus of this work.

These data provide important information that influences antisense drug discovery activities. We have previously reported that ASOs targeted to multiple repeats are more effective than those targeting a single site and that slowly spliced introns are more susceptible to ASO effects (9,32). In this study we provide for the first time, a semi-quantitative estimate of the time required for ASOs enter the cell, engage the cognate sequence in the target RNA and recruit RNase H1. In contrast to most small molecule drugs whose rates are diffusion limited (33), the rates of ASOs are much slower. Even cleavage of the target RNAs are relatively slow. Clearly, one reason that sites in introns are often less sensitive to ASO-induced cleavage is that spliced introns are cleaved during transcription, or as rapidly is the pre-mRNA is transcribed. These data once more emphasize the key roles played by levels of RNase H1 in cells and help explain the effects of alteration in splicing rates and multiple cognate sites in introns and exons on ASO activity. The rate limiting role of RNase H1, coupled to its low abundance and sequence preferences are surely significant contributors to the specificity of ASOs for the targeted sequence and limited off-target effects.

## Supplementary Material

SUPPLEMENTARY DATA
