# Supplementary material for: The rates of the major steps in the molecular mechanism of RNase H1-dependent antisense oligonucleotide induced degradation of RNA
Source: Nucleic Acids Res. 2015 Oct 10;43(18):8955–63. doi: 10.1093/nar/gkv920 (PMC4605327; doi:10.1093/nar/gkv920)
Supplement: SUPPLEMENTARY DATA [file supp_43_18_8955__index.html]

The rates of the major steps in the molecular mechanism of RNase H1-dependent antisense oligonucleotide induced degradation of RNA — The rates of the major steps in the molecular mechanism of RNase H1-dependent antisense oligonucleotide induced degradation of RNA — SUPPLEMENTARY DATA 

# The rates of the major steps in the molecular mechanism of RNase H1-dependent antisense oligonucleotide induced degradation of RNA

## SUPPLEMENTARY DATA

- SUPPLEMENTARY DATA
