# Supplementary material for: The rates of the major steps in the molecular mechanism of RNase H1-dependent antisense oligonucleotide induced degradation of RNA
Source: Nucleic Acids Res. 2015 Oct 10;43(18):8955–63. doi: 10.1093/nar/gkv920 (PMC4605327; doi:10.1093/nar/gkv920)
Supplement: SUPPLEMENTARY DATA [file supp_gkv920_nar-02028-y-2015-File008.pdf]

Figure S1. Copy number of minigene integrated in TREX 293 cells. Minigene copy number for SOD/TO or SOD/TO-187 cells was determined as detailed in Materials and Methods. For each cell line, four replicate CT values were obtained per with genomic DNA sample with FAM-labeled and VIC-labeled probes, assaying the promoter region of the pcDNA4/TO plasmid in which the minigene was cloned and an RNaseP control gene, respectively. Copy number was calculated by analysis of the PCR results with Copy Caller software.

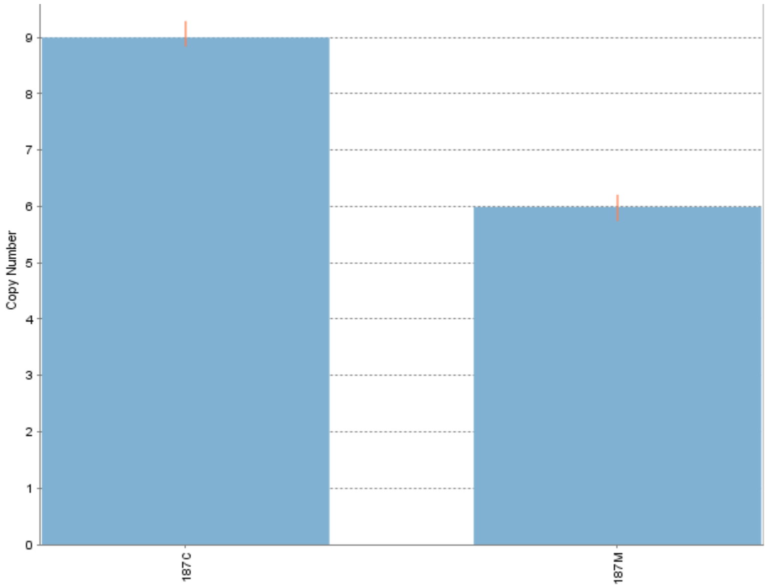

ample Nam	Reference	N Calculate	N Predicte	plicate Co	cates Anal	AM Ct Mea	/IC Ct Mea	elta Ct Mea	Delta Ct SD	elta Delta (	RQ	Min CN	Max CN	CN Range
SOD/TO	RNase_P	9.00	9	4	4	26.1048	27.1554	-1.0507	0.03	-2.17	4.50	8.84	9.29	0.45
187M	RNase_P	5.99	6	4	4	25.4475	25.9109	-0.4634	0.05	-1.58	3.00	5.74	6.21	0.47

Figure S2. SOD1 Minigene RNA half-life. SOD/TO or SOD187M/TO cells were seeded at 10,000 cells/well in 96 well plates then treated for 18 hours with 1 ug/ml TET to induce steady-state levels of the minigene transcript. Cells were washed extensively with PBS, then incubated in DMEM with 10% FCS and 5 ug/ml of Actinomycin D. Cells were harvested from 0-24 hours following the addition of Actinomycin D and total RNA purified. Minigene RNA levels were quantitated by qRT/PCR using primer/probe sets specific for the spliced or pre-mRNA. RNA levels are shown relative to T=0 for mRNA (A) or pre-mRNA (B) and are the mean of 4 replicate wells and normalized to total input RNA as determined by Ribogreen assay. SOD/TO cells, blue lines; SOD187M/TO cells, red lines

A)

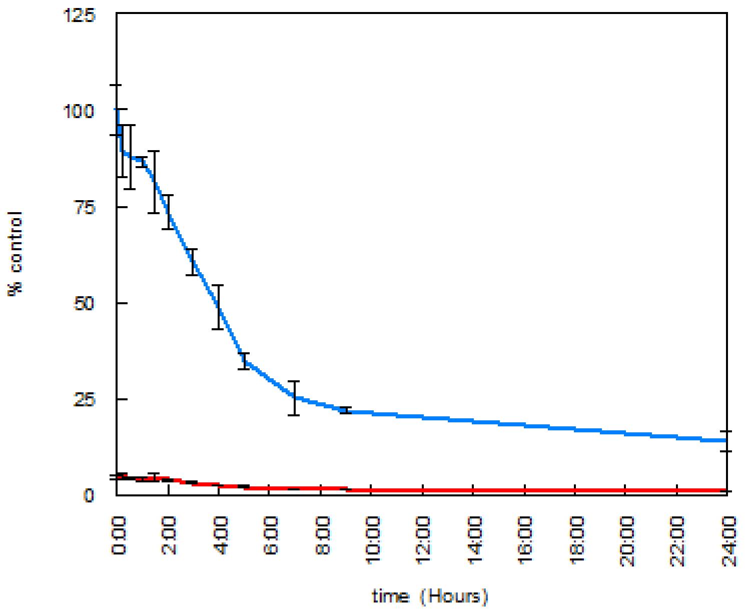

B)

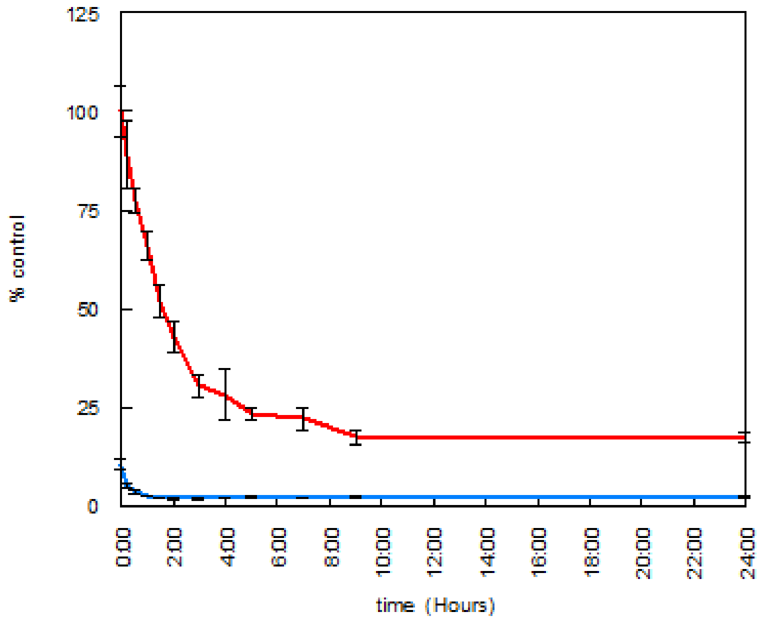

Figure S3.Transit times and target RNA degradation rates unaffected by ASO length or chemistry. HEK 293 cells harboring the SOD/TO minigene (solid lines) or the SOD/TO minigene and pcDNA3.1-RHA (+H1, dashed lines) were induced overnight with TET to establish steady-state RNA levels. TET was removed and cells washed extensively to stop transcription. Cells were immediately transfected with with a 5-10-5 MOE gap-mer ASO (MOE, blue) or with a 3-10-3 cEt gap-mer ASO (cEt, red) using Lipofectamine 2000, then harvested at various times after initiation of transfection with RLT lysis buffer and total RNA purified. Levels of total minigene RNA were assessed by qRT/PCR as described in Figure 6. A) Estimates of ASO transit and RNase H1 recruitment times. B) Data in panel A plotted as linear regression from 120-300 minutes for control and 40-120 minutes for cells overexpressing RNase H1.

A)

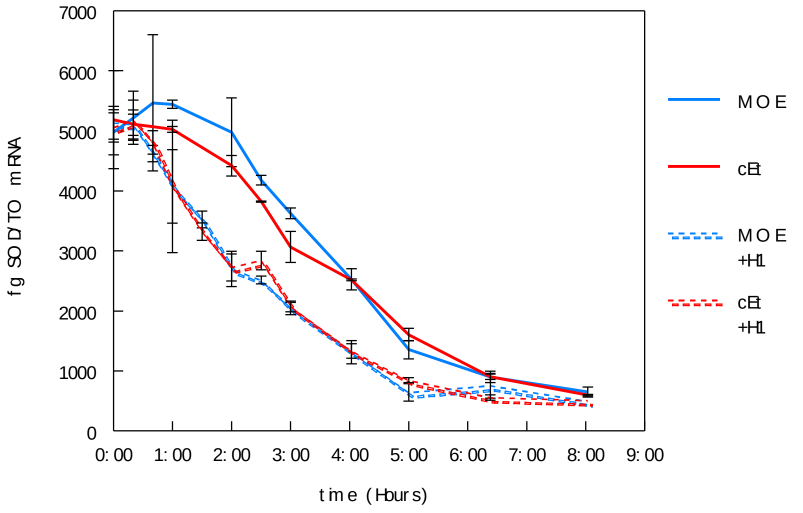

B)

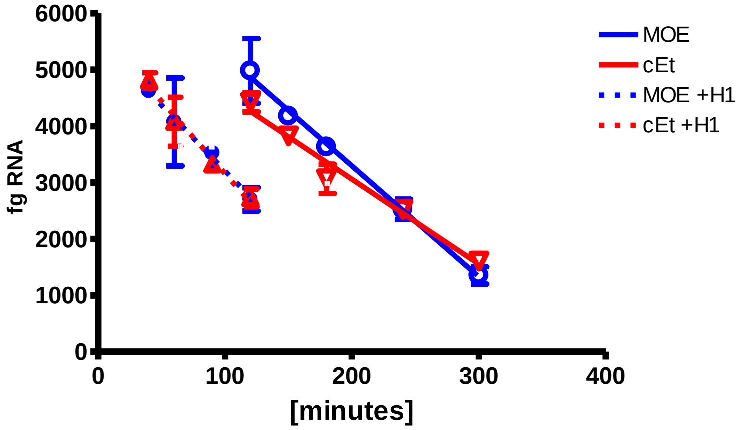

	MOE	cEt	MOE +H1	cEt +H1
Slope	-19.63 ± 0.6540	-15.04 ± 1.378	-23.35 ± 1.342	-25.65 ± 2.298
Y-intercept when X=0.0	7218 ± 136.2	6064 ± 286.9	5538 ± 111.7	5720 ± 191.3
X-intercept when Y=0.0	367.8	403.2	237.2	223.0
1/slope	-0.05095	-0.06649	-0.04283	-0.03898
95% Confidence Intervals				
Slope	-21.71 to -17.54	-19.42 to -10.66	-29.12 to -17.57	-35.54 to -15.76
Y-intercept when X=0.0	6785 to 7652	5151 to 6977	5058 to 6019	4897 to 6543
X-intercept when Y=0.0	350.3 to 389.2	354.0 to 490.6	204.8 to 290.4	181.3 to 315.5
Goodness of Fit				
r^2	0.9967	0.9754	0.9934	0.9842
